# The Impact of Climate Change and Environmental Stressors on Maternal Mental Health: A Narrative Review

**DOI:** 10.7759/cureus.88519

**Published:** 2025-07-22

**Authors:** Aditi Das, Monalisa Mall, Barsharani Behera, Rojalin Dash, Bishnupriya Lenka, Swagata Sahoo

**Affiliations:** 1 Obstetric and Gynaecological Nursing, Kalinga Institute of Nursing Sciences, Kalinga Institute of Industrial Technology, Deemed to be University (KIIT-DU), Bhubaneswar, IND; 2 Obstetrics and Gynaecology, Kalinga Institute of Nursing Sciences, Bhubaneswar, IND; 3 Obstetrics and Gynaecology, Kalinga Institute of Nursing Sciences, Kalinga Institute of Industrial Technology, Deemed to be University (KIIT-DU), Bhubaneswar, IND; 4 Obstetrics and Nursing, Kalinga Institute of Nursing Sciences, Bhubaneswar, IND; 5 Nursing, Kalinga Institute of Nursing Sciences, Bhubaneswar, IND; 6 Obstetric and Gynaecological Nursing, Kalinga Institute of Industrial Technology, Deemed to be University (KIIT-DU), Bhubaneswar, IND

**Keywords:** climate change, environmental stressor, maternal mental health, perinatal period, postpartum depression

## Abstract

Climate change presents an urgent and growing threat to global health, with particularly profound implications for maternal mental health. Pregnant and postpartum women are uniquely vulnerable to climate-related stressors due to physiological, psychological, and social sensitivities during the perinatal period. However, this intersection remains critically underexplored in public health research and policy. This narrative review critically examines and synthesizes the emerging evidence on the impact of climate-related environmental stressors, including extreme heat, air pollution, natural disasters, food insecurity, and displacement, on maternal mental health outcomes. It explores how these stressors contribute to increased risks of perinatal mood and anxiety disorders, postpartum depression, and trauma-related symptoms. A structured literature search of PubMed and Web of Science identified 33 high-quality studies published between 2012 and 2025, which were analyzed to identify patterns, gaps, and key mechanisms of vulnerability. Findings reveal that climate change exacerbates maternal mental health risks through interrelated pathways involving direct environmental exposures, disrupted access to care, psychosocial stress, and systemic inequities. Climate change adversely affects fetal outcomes through heat exposure, air pollution, natural disasters, and environmental toxins, increasing risks of preterm birth, low birthweight, stillbirth, and impaired child development. Despite growing recognition of these links, maternal mental health remains insufficiently integrated into climate resilience planning and healthcare systems. Addressing this gap requires an interdisciplinary, equity-focused approach that embeds maternal well-being within climate adaptation strategies. Proactive, inclusive policies and interventions are essential to mitigate emerging threats and promote resilience for mothers, families, and communities in the face of rapidly changing climate.

## Introduction and background

Climate change poses a rapidly intensifying threat to global health, with disproportionate impacts on vulnerable populations, including pregnant and postpartum women. As environmental stressors, such as extreme heat, natural disasters, air pollution, food insecurity, and displacement, become more frequent and severe, their implications for maternal mental health demand urgent attention. The perinatal period is a time of heightened physiological and psychological vulnerability. Disruptions during this critical window may not only affect maternal well-being but also have lasting consequences for offspring development and family dynamics [1-3].

Emerging research demonstrates associations between climate-related events and increased risk of perinatal mood and anxiety disorders (PMADs), postpartum depression, and heightened psychological distress [4-6]. Acute exposures to high ambient temperatures have been linked to increased psychiatric emergency department visits among pregnant populations, with notable surges in anxiety and suicidal ideation [7]. Qualitative and quantitative findings from diverse global contexts further reveal how environmental stressors, such as extreme weather events (EWEs), exacerbate maternal trauma, social isolation, economic strain, and feelings of helplessness [8-10].

Despite the growing body of evidence, the intersection of climate change and maternal mental health remains under-researched and under-prioritized in public health policy. Climate-related psychological burdens, such as "eco-anxiety" and “climate grief”, are increasingly recognized, yet their specific impact on maternal-fetal bonding, antenatal anxiety, and postpartum adjustment remains insufficiently addressed [11-13]. Studies from Australia, the United States, Africa, and the Middle East highlight a global concern, but also reveal regional disparities in risk exposure, healthcare access, and adaptive capacity [6,9,14,15].

This narrative review highlights how climate change and related environmental stressors, such as extreme heat, air pollution, natural disasters, and food insecurity, adversely affect maternal mental health. Framed within an equity-based perspective, the review identifies key vulnerabilities, including physiological sensitivity during pregnancy and systemic barriers to care. It emphasizes the urgent need for coordinated clinical, policy, and research strategies to address these risks and strengthen resilience within maternal-child health systems worldwide.

## Review

Search methodology

The search methodology for this narrative review followed a comprehensive and structured approach to identify relevant literature. The review utilized two commonly used electronic databases, PubMed and Web of Science. Search terms included “climate change,” “environmental stressor,” and “maternal mental health,” along with related keywords, combined using Boolean operators to refine and optimize search results.

This review focused on studies examining the impact of climate change, environmental stressors, and maternal mental health. The included studies involved pregnant or postpartum women exposed to factors such as extreme weather, air pollution, and natural disasters, with reported outcomes, including depression, anxiety, post-traumatic stress disorder (PTSD), or stress-related symptoms. Eligible studies included observational research, clinical studies, reviews, and qualitative analyses published in English from 2012 to 2025. A total of 125 articles were identified, of which 33 met inclusion criteria based on relevance, quality, and methodological rigor. These studies analyzed the impact of climate change and environmental risk factors on maternal psychological health.

Climate change and environmental stressors

Climate change poses significant risks to maternal mental health, primarily through environmental exposures. Extreme heat increases the risk of perinatal mood disorders and psychiatric emergencies, particularly among marginalized groups [16,17]. Poor air quality, often exacerbated by wildfires and pollution, is linked to heightened anxiety and depression during pregnancy [3]. Food insecurity, worsened by climate disruptions, contributes to maternal stress and postpartum depression, especially in low-resource settings [14]. Exposure to indoor pollutants, such as organophosphate esters (OPEs), and heavy metals, like cadmium and mercury, further elevates psychological distress and affects infant neurodevelopment [13,18,19]. Traumatic climate events, floods, hurricanes, and wildfires, are associated with long-term stress, social isolation, and post-traumatic symptoms in mothers [4,5]. Climate change acts as a significant environmental health stressor by intensifying these factors, disproportionately affecting maternal mental health, especially in low- and middle-income countries [20].

Maternal mental health: overview

Climate change presents a growing and multifaceted threat to maternal mental health, particularly during pregnancy and the postpartum period. Rising global temperatures, extreme weather events, and environmental degradation have been associated with heightened risks of anxiety, depression, and post-traumatic stress among mothers [2,5,6]. Disasters such as wildfires and floods disrupt social support systems, exacerbate pre-existing mental health conditions, and hinder access to healthcare and essential resources, thereby contributing to food insecurity and psychological distress [8,9,12]. Forced migration and sociopolitical instability, frequently driven by climate-related events, impose additional burdens on maternal and child health [13]. Prenatal exposure to environmental trauma, including natural disasters and chemical toxicants, has demonstrated both physiological and psychological consequences for the mother and fetus, including alterations in placental function and infant temperament [21-24]. Moreover, climate anxiety has been associated with increased antenatal stress and shifting reproductive intentions, including the emergence of antinatalist sentiments [25,26]. These compounded stressors are especially pronounced in low- and middle-income countries, where systemic inequities amplify maternal vulnerability [27,28]. Heatwaves and wildfires have been shown to adversely affect maternal health and neonatal outcomes, underscoring the need for urgent measures to assess and mitigate exposure during pregnancy [29]. Qualitative evidence drawn from women’s lived experiences under extreme heat and climate-related stress highlights daily coping challenges, particularly within climate-vulnerable communities [30,31]. Climate-sensitive diseases, such as vector-borne infections, waterborne illnesses, and heat-related conditions, are exacerbated by climate change and disproportionately affect pregnant women, thereby increasing both physical and psychological vulnerability [32]. Collectively, these intersecting factors contribute to heightened maternal mental health risks, including anxiety, depression, and emotional distress, particularly in low-resource and climate-impacted settings [33].

Interplay between climate change and maternal mental health

Psychosocial and Emotional Impacts

Climate change significantly contributes to psychosocial stress among pregnant and postpartum women through both direct and indirect emotional pathways. Emotional responses, such as ecological grief, fear of environmental decline, and climate-related anxiety, can interfere with maternal-fetal bonding and heighten antenatal stress, particularly among primigravida women [2]. Psychological trauma resulting from extreme weather events (EWEs), including floods and droughts, often leads to long-term emotional distress, feelings of helplessness, and social isolation, which undermine maternal coping capacities [3,4].

Prenatal exposure to elevated temperatures and air pollution has been associated with adverse birth outcomes, including preterm birth and low birth weight, which in turn amplify maternal psychological distress during and after pregnancy [6,17]. In rural Uganda, climate-induced food insecurity exacerbated by unpredictable weather patterns has been identified as a key factor contributing to perinatal mood and anxiety disorders, demonstrating how environmental hardship triggers emotional suffering during pregnancy [33].

Additionally, sociocultural stressors, such as discrimination and acculturative pressure, intersect with climate vulnerability to intensify maternal mental health challenges. This is evident in studies of Latina mothers in the U.S., who reported increased psychological distress amid sociopolitical instability compounded by environmental stressors [25]. Temperature extremes and poor air quality have also been linked to heightened postpartum depressive symptoms, particularly among populations with limited access to environmental protections like green spaces or cooling systems [30]. Collectively, these psychosocial and emotional impacts highlight the complex and multifaceted ways in which climate change amplifies maternal mental health vulnerabilities across diverse geographic, socioeconomic, and cultural contexts.

Health Systems Disruptions and Intergenerational Consequences

Prenatal maternal stress associated with natural disasters has been demonstrated to adversely impact offspring neurodevelopment across the cognitive, motor, socio-emotional, and behavioral domains, highlighting the profound intergenerational consequences of climate-related maternal mental health challenges [20]. In low- and middle-income countries, climate-induced disruptions to healthcare systems during extreme weather events (EWEs) exacerbate these challenges by restricting access to essential maternal and perinatal services. Such disruptions contribute to increased maternal mortality and heightened risks of malnutrition, thereby amplifying psychological distress during pregnancy [12]. These findings underscore the urgent need for multi-level, integrated strategies that simultaneously address environmental exposures, psychosocial stressors, and systemic healthcare vulnerabilities to protect maternal and child health in the context of a changing climate.

Effect of Climate Change on Fetal Outcomes

Climate change poses significant risks to fetal health through direct and indirect pathways. Exposure to extreme heat, wildfire smoke, and natural disasters increasingly contributes to adverse birth outcomes such as preterm birth, low birthweight, and stillbirth. For example, heatwave days are associated with a 16% increase in preterm birth and a 46% increase in stillbirth risk, with each additional degree Fahrenheit raising these risks by 5% [34]. Similarly, wildfire smoke exposure during pregnancy increases preterm birth risk by approximately 0.5% per day of exposure [35]. Prenatal maternal stress caused by climate-related disasters has also been linked to impaired child development and health [12,24]. Environmental contamination exacerbated by climate change leads to prenatal exposure to dietary toxicants and heavy metals, which negatively affect infant neurodevelopment [13,19]. While residential greenery may offer protective benefits for pregnancy outcomes [36], systemic challenges like food insecurity and limited healthcare access compound vulnerabilities [37]. Recognizing these multifaceted threats, global health authorities emphasize integrating maternal and child health into climate policy and response frameworks to safeguard fetal health in a changing climate (Table 1) [31,38].

**Table 1:** Features of reviewed articles

Author	Year	Key Findings	Conclusion
Dadvand P et al. [36]	2012	Green surroundings during pregnancy linked to better birth outcomes.	Urban green spaces benefit maternal and child health.
Nomura Y et al. [10]	2019	Prenatal exposure to disaster-related maternal depression affects infant temperament by 6 months.	Prenatal disaster stress has lasting effects on infant development.
Pardhi A et al. [23]	2020	Migrant mothers face poor healthcare access, compounded by displacement stress.	Holistic, migrant-sensitive maternal services needed.
Chersich MF et al. [34]	2020	High temperatures linked with increased risk of preterm birth, low birth weight, and stillbirth.	Protecting pregnant women from heat exposure is critical.
Lafortune S et al. [12]	2021	Maternal stress due to disasters negatively impacts child health and development.	Disaster-related prenatal stress leads to developmental risks; early interventions are crucial.
Mark TE et al. [14]	2021	Food insecurity during seasonal shifts correlates with postpartum depression in rural Malawi.	Seasonal and climate-related food scarcity worsens maternal mental health.
Jain et al. [37]	2021	Environmental stressors increase adverse pregnancy outcomes; OPERA program offers early screening and intervention.	Preventive strategies like OPERA can reduce risks in vulnerable populations.
Heft-Neal S et al. [35]	2021	Wildfire smoke during pregnancy associated with preterm birth in California.	Air quality monitoring and maternal advisories are essential.
Buthmann J et al. [24]	2022	Climate-related disaster alters placental gene expression and infant temperament.	Environmental stress affects fetal development biologically.
Non AL et al. [25]	2022	Latina mothers’ mental health worsened with sociopolitical and climate-related stress.	Mental health services must consider sociocultural stressors.
Karuga FF et al. [26]	2022	Climate anxiety and poor obstetric care linked to antinatalism in Poland.	Mental health and reproductive choices are shaped by environmental fears.
Rothschild J, Haase E [3]	2023	Climate change causes direct neuropsychiatric effects like trauma, stress, and emotional dysregulation in women.	Women’s mental health must be prioritized in climate policies.
Fan WN, Zlatnik MG [6]	2023	Climate change threatens pregnancy through heat, food insecurity, and displacement.	Need for mitigation, adaptation, and resilience in maternal care.
Pandipati S, Abel D [28]	2023	Women face disproportionate health risks from climate impacts due to biology and social roles.	Gender-sensitive climate policies are crucial.
Bansal A et al. [29]	2023	Heatwaves and wildfires impair maternal health and neonatal outcomes.	Calls for urgent action to assess and reduce exposure during pregnancy.
Amin SM et al. [2]	2024	Emotional responses to climate change are associated with antenatal anxiety and weaker maternal-fetal attachment.	Psychosocial care must consider climate-related anxieties.
Pardon MK et al. [4]	2024	Extreme weather (e.g., floods) increases PTSD, anxiety, and depression in mothers.	Disaster preparedness should include maternal mental health.
Barkin JL et al. [5]	2024	Perinatal women are at increased risk, yet climate threats remain underrecognized in psychiatric care.	More research and policy efforts needed in perinatal mental health and climate intersections.
Runkle JD et al. [7]	2024	Elevated temperatures during pregnancy correlate with higher psychiatric emergency visits.	Temperature monitoring should guide mental health interventions.
Kadio K et al. [8]	2024	Extreme heat negatively impacts pregnant women’s well-being in Burkina Faso.	Community-based, locally adapted support is essential.
Kou XR et al. [13]	2024	Prenatal exposure to dietary toxicants like mercury from fish affects infant neurodevelopment.	Climate-sensitive dietary practices are needed during pregnancy.
Sun Y et al. [17]	2024	High postpartum temperatures associated with increased risk of postpartum depression.	Climate-sensitive postpartum care models are needed.
Foster SA et al. [18]	2024	Exposure to organophosphates and plasticizers during pregnancy linked to maternal anxiety and depression.	Household chemical exposures should be regulated for maternal well-being.
Buchwald AG, Boudova S [20]	2024	Emphasizes maternal mental health effects in low-income countries are often overlooked.	Climate and mental health responses must be globally inclusive.
Pappas A et al. [30]	2024	In LMICs, extreme weather disrupts maternal care and increases mental health burdens.	Infrastructure and maternal health resilience must be built in LMICs.
Bryson JM et al. [33]	2024	Ugandan women report stress due to food insecurity driven by climate change during pregnancy.	Food security and maternal mental health are interconnected.
Barkin JL et al. [1]	2025	Climate change-related stressors (heatwaves, pollution) increase risk of perinatal depression and anxiety.	Climate change is a critical factor affecting perinatal mental health; integrated responses needed.
de Cuba SE et al. [9]	2025	Immigrant mothers experience added stress due to climate vulnerability and health system barriers.	Policies must address socio-environmental disparities.
Ulrich SE et al. [16]	2025	Maternal mental health disparities were evident post-heatwave; Black and low-income women were more affected.	Equity-based heat response strategies are essential.
Kou XR et al. [19]	2025	Prenatal exposure to heavy metals adversely affects infant neurodevelopment.	Reducing environmental pollutants during pregnancy is essential.
Amekpor F et al. [31]	2025	Proposes integrating maternal-child health into climate policies through a holistic model.	Holistic planning enhances resilience and health equity.
Kabir MI et al. [32]	2025	Systematic review shows climate-sensitive diseases affecting maternal health in Bangladesh.	Data-driven policymaking is needed in climate-vulnerable nations.
Kaya et al. [27]	2025	Climate change education reduced anxiety and improved awareness among pregnant women.	Education is effective in improving maternal mental health and climate literacy.

Implications and future directions

Emerging evidence elucidating the relationship between climate change, environmental stressors, and maternal mental health signals a critical and escalating public health challenge with significant ramifications for both maternal and child outcomes. This narrative review highlights the complex, multifactorial nature of climate-related risks, encompassing direct environmental exposures, physiological susceptibilities, and intricate psychosocial dynamics. Effectively mitigating these impacts necessitates a concerted, interdisciplinary approach that bridges scientific inquiry, clinical intervention, and policy development to protect and promote maternal mental well-being in the context of a rapidly changing climate.

Recommendations

The global climate crisis presents a critical threat to maternal mental health, particularly among vulnerable populations in low-resource and marginalized settings. A comprehensive, equity-focused response is essential to mitigate adverse outcomes and build resilience. Future interdisciplinary research should examine the links between climate exposures, such as extreme heat and air pollution, and maternal mental health, incorporating both biomarker data and psychosocial assessments. Integrating climate-related risks into perinatal mental health screenings can facilitate early identification of stressors like climate anxiety and eco-grief.

**Figure 1:**
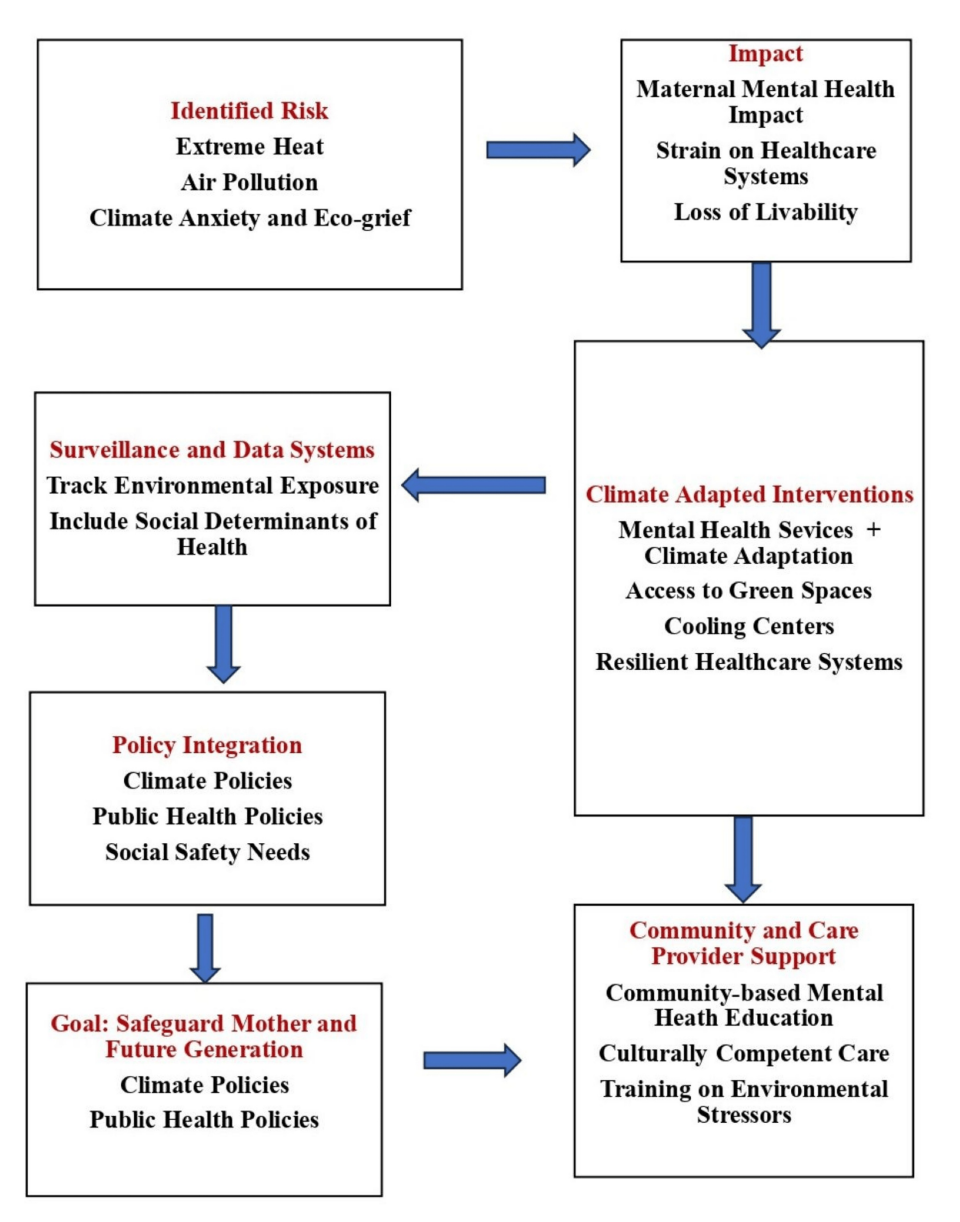
Recommendations for future action

Enhanced surveillance systems that include environmental exposures and social determinants of health are needed to inform predictive analytics and optimize interventions. Strategies should align mental health services with climate adaptation, ensuring access to green spaces, cooling resources, and resilient healthcare infrastructure. Community-based psychoeducation, culturally competent care, and provider training on environmental stressors are vital. Embedding maternal mental health into climate and public health policies, with robust social safety nets, will be key to safeguarding the well-being of mothers and future generations. In addition, obstetricians, midwives, and primary care clinicians can play a key role by incorporating climate-related stressors into routine maternal assessments, educating patients about environmental health risks, advocating for local climate resilience efforts, and connecting at-risk individuals to mental health and social support services. Their proactive engagement can enhance early detection, reduce disparities, and promote resilience at the front lines of care (Figure 1).

## Conclusions

Climate change represents a complex and escalating threat to maternal mental health, particularly for populations that are socioeconomically disadvantaged and environmentally at risk. This review emphasizes the urgent need for integrated, interdisciplinary strategies that address the physiological, psychological, and social pathways through which climate-related stressors lead to negative maternal outcomes. Current evidence highlights the substantial impact of extreme heat, air pollution, natural disasters, and environmental toxins on perinatal mental health, which can have far-reaching effects on child development and family well-being. Effective mitigation of these risks requires prioritizing longitudinal, equity-focused research, alongside the integration of maternal mental health into healthcare systems and climate policy frameworks. Proactive, inclusive, and interdisciplinary approaches are essential to safeguard maternal well-being amid growing climate instability.
